# Co‐Infection With Hepatitis E Virus and SARS‐CoV‐2 in a Lymphoma Patient

**DOI:** 10.1002/jmv.70706

**Published:** 2025-11-18

**Authors:** Yacine Boucetta, Céline Boschi, Erika Peroni, Rodolphe Jean, Erwan Bories, Patrick Borentain, Julien Andreani, Philippe Colson

**Affiliations:** ^1^ IHU Méditerranée Infection Marseille France; ^2^ Assistance Publique‐Hôpitaux de Marseille (AP‐HM) Marseille France; ^3^ Aix‐Marseille Univ., Microbes Evolution Phylogeny and Infections (MEPHI) Marseille France; ^4^ Assistance Publique‐Hôpitaux de Marseille (AP‐HM), Internal Medicine and Clinical Immunology Department CHU Conception Marseille France; ^5^ Private medical practice in hepatogastroenterology Aix‐en‐Provence France; ^6^ Assistance Publique‐Hôpitaux de Marseille (AP‐HM), Hôpital Timone, service d'Hépatologie Marseille France

**Keywords:** coinfection, hepatitis, hepatitis E virus, SARS‐CoV‐2

## Abstract

Hepatitis E virus (HEV) and SARS‐CoV‐2 are both ubiquitous worldwide. We report here a rare case of HEV and SARS‐CoV‐2 coinfection. HEV and SARS‐CoV‐2 diagnoses were carried out by qPCR. Virus genotyping was performed by analyzing partial or near full‐length genomes obtained by next‐generation sequencing from plasma or nasopharyngeal specimens, respectively. The case was a lymphoma patient admitted to hospital for oxygen‐requiring qPCR‐documented Covid‐19. SARS‐CoV‐2 was a Delta variant. Reevaluation of a liver cytolysis documented 1 year earlier revealed concurrent chronic hepatitis E at cirrhosis stage. HEV genotype was 3c. Ribavirin was administered, but to date only temporarily led to HEV RNA undetectability in plasma when combined with immunosuppressive therapy interruption. This case and the three previous cases of coinfection with HEV and SARS‐CoV‐2 point out the possible deleterious effect of SARS‐CoV‐2 on the liver and the increased risk of such concurrent infections in immunocompromized patients.

## 1 Introduction

Hepatitis E virus (HEV) and severe acute respiratory syndrome coronavirus 2 (SARS‐CoV‐2) are both single‐stranded (+) RNA viruses. HEV was discovered in 1983 in Russia and SARS‐CoV‐2 in 2019 in China [1, 2]. Both are ubiquitous worldwide. HEV is a major cause of acute hepatitis, and its epidemiology varies according to the geographical area and the genotype as it is mainly waterborne in developing countries and most often documented as associated with consumption of pig‐derived products in developed countries [2]. It can cause chronic hepatitis and cirrhosis in immunocompromised people. It is endemic in the south of France [3]. SARS‐CoV‐2 became pandemic during early 2020 and essentially causes a respiratory syndrome, COVID‐19, which can be clinically severe [4, 5]. Due to the tremendous SARS‐CoV‐2 incidence at the global and country scales over the past 5 years, many concurrent infections occurred in SARS‐CoV‐2‐positive patients. These included infections with other respiratory viruses [6] but also with a broad range of other viruses and microorganisms. Here we report a case of co‐infection with HEV and SARS‐CoV‐2 in an immunocompromised patient with severe pneumonia.

## 2 Case Report

A man in his fifties with marginal zone lymphoma initially treated with Ibrutinib and Human immunoglobulins was hospitalized during fall 2021 for oxygen‐dependent pneumonia due to SARS‐CoV‐2. First symptoms appeared 5 days before admission and included cough, asthenia, right ear pain and fever. The patient was diagnosed as infected by SARS‐CoV‐2 using a real‐time PCR (qPCR) assay (BGI, Shenzhen, China), qPCR cycle threshold value (Ct) being 20. A Delta variant was identified using the Nextclade web application (https://clades.nextstrain.org/) [7] based on the viral genome (GenBank (https://www.ncbi.nlm.nih.gov/genbank/) Accession no. ON276954.1) obtained by next‐generation sequencing (NGS) with the Illumina technology (Illumina Inc., San Diego, USA) on a NovaSeq 6000 Instrument as previously described [8]. The patient had received two doses of the Pfizer‐BioNTech COVID‐19 mRNA vaccine 3 and 5 months before this episode, as well as immunoglobulins (Polyvalent human immunoglobulins, 30 mg) due to hypogammaglobulinemia at 1.63 g/L. Because of hypoxia and initial pulmonary damage estimated at 50%–60% by computed tomography scan, he received oxygen therapy at 8 L/min. Probabilistic antibiotic therapy with ceftriaxone was implemented for 5 days and switched to piperacillin/tazobactam (4 g, three times a day) for 5 days combined with dexamethasone (6 mg/day), anakinra (300 mg), and an injection of the combination of casirivimab‐imdevimab, two monoclonal antibodies. Microbial cultures of blood and urine returned negative. SARS‐CoV‐2 serology (Liaison XL DiaSorin, Saluggia, Italy) was weakly positive (67 binding antibody units (BAU)/mL) 5 days post‐admission. SARS‐CoV‐2 qPCR turned negative on a nasopharyngeal specimen swab and weaning from oxygen therapy occurred 5 days later.

**Figure 1:**
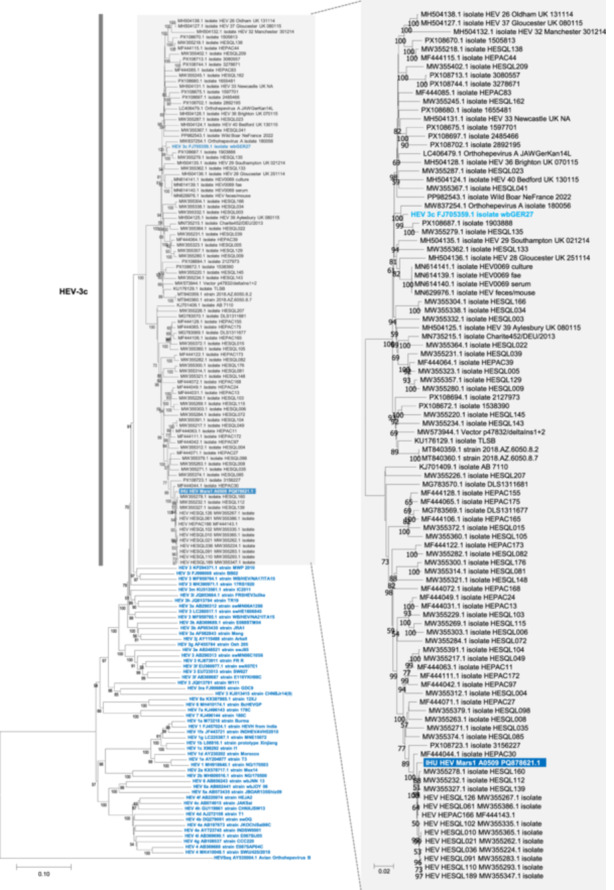
Phylogenetic tree that incorporated the partial HEV genome of the index case. The HEV partial genome sequence obtained by next‐generation sequencing directly from the blood sample through metagenomics from the case is indicated by a white bold font and a dark blue background. It spans positions 86‐7,017 from genome GenBank Accession no. FJ705359. The 100 sequences with the highest BLAST (https://blast.ncbi.nlm.nih.gov/Blast.cgi) scores recovered from the NBCI GenBank nucleotide sequence databases (http://www.ncbi.nlm.nih.gov/nucleotide/), were incorporated in the phylogeny reconstruction, in addition to reference sequences for HEV genotypes, indicated by a light blue bold font [10]. Nucleotide alignments were performed using the Mafft web application (https://mafft.cbrc.jp/alignment/server/index.html). Avian orthohepevirus was used as outgroup sequence. Phylogeny reconstruction was carried out using the MEGA v11 program (https://www.megasoftware.net/). The evolutionary history was inferred using the Neighbor‐Joining method. The evolutionary distances were computed using the Kimura 2‐parameter method and are in the units of the number of base substitutions per site. The percentage of replicate trees in which the associated taxa clustered together in the bootstrap test (1,000 replicates) are shown below the branches. Bootstrap values > 50% are labeled on the tree. The tree is drawn to scale, with branch lengths in the same units as those of the evolutionary distances used to infer the phylogenetic tree; the scale bars indicate the number of nucleotide substitutions per site.

In parallel with his respiratory infection, the patient was presenting a perturbation of liver biochemical tests with a cytolysis and anicteric cholestasis the day of his admission (alanine aminotransferase (ALT), 98 IU/L; aspartate aminotransferase (AST), 138 IU/L; gammaglutamyltransferase (GGT), 376 IU/L; total bilirubinemia 23 µmol/L). As a matter of fact, liver disturbances were already present in 2020, and liver biopsy performed during summer 2021 showed fibrosis at METAVIR stage F3. Two months before admission, ALT, AST and GGT were 444, 269, and 293 IU/L, respectively, and shear wave elastography showed liver fibrosis at METAVIR stage F4 (13 kPa), indicating liver cirrhosis. Autoimmune hepatitis had been considered but auto‐antibodies were negative, while liver toxicity of ibrutinib was also suspected. At the time SARS‐CoV‐2 qPCR turned negative, HEV qPCR was performed and found positive, viral load being 7.5 log_10_IU/mL in plasma and 8.3 log_10_IU/mL in feces (Altona diagnostics GmbH, Hamburg, Germany; detection threshold, 2.3 log_10_IU/mL). Anti‐HEV IgG (Wantai, Beijing China) and IgM (Liaison XL, DiaSorin) testing were negative. HEV genotype was 3c, as determined by a BLAST search and a phylogenetic analysis based on the partial viral genome (GenBank no. PQ878621.1) obtained directly from the DNA/RNA extract recovered from the plasma by NGS using the Illumina technology, as previously described [9] (Figure 1). No source or transmission route of HEV infection was documented.

One week after the HEV diagnosis, plasma HEV RNA load was still 7.7 log_10_IU/mL, but the patient had improved clinically and returned home. Due to the patient's immunocompromised status and the at least 3 month‐duration of the liver cytolysis, treatment with ribavirin (800 mg per day) was introduced for 3 months. Plasma HEV RNA was still detectable (5.4 log_10_IU/mL) after this 3‐month course of therapy but ribavirin was nonetheless discontinued for 3 months due to drug shortage. Resumption of ribavirin (800 mg per day) occurred at month 7, combined with the cessation of ibrutinib immunosuppressive therapy at month 9. This led to HEV RNA undetectability at month 10 of first ribavirin introduction. Ribavirin was discontinued, but a virological rebound occurred 4 months later. Ribavirin was reintroduced at month 18 of first ribavirin introduction, at dosages of 800 mg per day, then 1200 mg per day and 400 mg per day for 6, 6 and 10 months, respectively. From month 21 through month 32 of first ribavirin introduction, HEV RNA was tested on five occurrences in plasma and was detectable in all cases, with viral loads ranging between 1.9 and 2.4 log_10_IU/mL. Thereafter, the patient was lost to follow‐up.

## 3 Discussion

To our best knowledge, the present case is the fourth report of HEV and SARS‐CoV‐2 co‐infection [11, 12]. The two first cases (both men in their 60 s) were reported to result from nosocomial transmission, documented by sequence analysis, of a HEV of genotype 3 f in an intensive care unit in England in 2021 [11] (Table 1). This was possibly due to a failure in infection control practices in the context of the Covid‐19 pandemics with sharing of a two‐bed space. The deemed source patient, with a medical history of ischemic heart disease, hypertension and alpha‐thalassemia, experienced chronic HEV infection requiring ribavirin therapy in the setting of immunomodulation by dexamethasone and tocilizumab for his persistent SARS‐CoV‐2 infection. The exposed patient, with a medical history of hypertension, asthma, obstructive sleep apnea and benign prostatic hyperplasia, experienced HEV infection that resolved spontaneously in approximately 1 month [11]. The third case, described in China, was a man in his 60 s who was immunocompromised due to chronic lymphocytic leukemia [12]. He was concomitantly diagnosed with SARS‐CoV‐2 and HEV infections by molecular testing, and SARS‐CoV‐2 replication and related symptoms persisted for approximately 60 days, with administration of methylprednisolone and lately, nirmatrelvir/ritonavir. HEV RNA testing was performed again 1 month post‐discharge and was negative. Overall, these cases highlight the increased risk of chronic HEV infection in patients with a weakened immune status in the setting of anti‐SARS‐CoV‐2 therapies, and include one of the rare cases of HEV human‐to‐human transmission.

As a matter of fact, there is evidence of the deleterious effect of SARS‐CoV‐2 infection on the liver [13, 14, 15]. Thus, a Chinese study conducted in 2020–2021 identified that 18% of 7,622 Covid‐19 patients experienced a rise of ALT/AST, with two patterns observed including an early one within 14 days of symptom onset and a late one more than 14 days from symptom onset [16]. Such liver perturbations were significantly associated with a lower SARS‐CoV‐2 qPCR Ct and a longer duration of SARS‐CoV‐2 RNA‐positivity, and this suggested a potential direct virus injury to the liver. In another study conducted in 13 Asian countries, among SARS‐CoV‐2 infected patients, 43% of those with chronic liver disease developed acute liver injury and 20% of those with cirrhosis developed acute‐on‐chronic liver failure (11%) or acute decompensation (9%) [17]. In addition, liver injury was progressive in 57% of patients with decompensated cirrhosis, with 43% mortality. In an Italian multicenter retrospective study, of 50 SARS‐CoV‐2‐infected patients with cirrhosis, acute‐on‐chronic liver failure and *de novo* acute liver injury occurred in 14 (28%) and 10 (20%) patients, respectively [18]. Also, in a German cohort of 72 SARS‐CoV‐2‐infected patients, AST and ALT were increased in 63% and 39% of the cases, while these proportions were 57% and 37%, respectively in a US cohort of 1,219 SARS‐CoV‐2‐infected patients [14]. In addition, Wanner et al also provided evidence by multiple approaches of SARS‐CoV‐2 tropism to the liver, with viral RNA detection in approximately two‐thirds of autopsy liver specimens from patients who exhibited severe COVID‐19 and isolation of infectious virus from liver tissue post‐mortem. Moreover, they reported in some cases transcriptional and proteomic signatures in liver autopsy samples that were similar to the signatures associated with multiple other viral infections of the human liver, which included significant upregulation of type I and II interferon responses, IFN‐related JAK‐STAT signaling, and liver‐specific metabolic modulation [14]. Recently, SARS‐CoV‐2 spike and nucleocapsid proteins were detected in liver tissues from SARS‐CoV‐2‐infected patients with severe disease or deceased, and spatial transcriptomics revealed that SARS‐CoV‐2 RNA was also present in liver tissues [19]. Besides, SARS‐CoV‐2 was reported to bind to the ACE2 receptor on hepatocytes and cholangiocyte membrane [20]. It is also worthy to note that SARS‐CoV, which caused an outbreak of severe acute respiratory diseases in 2003 in Asia, was reported to be associated with hepatitis and was detected in liver tissue samples [15, 21]. In the present case, the patient presented a liver cytolysis at approximately 10 times the upper usual values 2 months before HEV diagnosis. Autoimmune hepatitis, which was associated with a bad clinical outcome in a large Covid‐19 series [22], was suspected although not ascertained.

Overall, previous data highlight that protracted HEV and SARS‐CoV‐2 infections that occur in immunocompromised patients increase the risk of co‐incidence of these infections that are both quite common worldwide and in Europe. Consequently, HEV infections, acute or chronic, may have been overlooked during the SARS‐CoV‐2 pandemic. Testing for HEV in case of liver cytolysis may be warranted regardless of another or other possible etiologies, including SARS‐CoV‐2.

**Table 1:** Main characteristics of cases of HEV and SARS‐CoV‐2 co‐infection.

Case no., reference	Country, year	Gender, age	Underlying conditions	HEV diagnosis	HEV source	Covid‐19 therapy	Hepatitis E therapy	Outcome
1 [11],	United Kingdom, 2021	M, 69	High blood pressure, asthma, obstructive sleep apnea, benign prostatic hyperplasia	On day 111 of admission for Covid‐19. HEV IgG negative, IgM positive, HEV RNA = 6.0 log_10_IU/mL. Retrospective testing 35 days earlier: Positive HEV RNA test	Unknown; Ate pork	High flow oxygen, remdesivir, dexamethasone, tocilizumab, amoxicillin/clavulanic acid	None	Covid‐19: Transferred in ICU 6 days post‐admission; transferred to medical ward on day 72. HEV: HEV RNA in serum peaked at > 6.7 log_10_IU/mL then felt to 3.6 log_10_IU/mL IU/mL on day 123; complete resolution
2 [11],	United Kingdom, 2021	M, 69	Ischemic heart disease, high blood pressure, alpha‐thalassemia	On day 13 of admission for Covid‐19. Blood: HEV IgG negative, IgM indeterminate, HEV RNA > 6.7 log_10_IU/mL; Feces: > 6.7 log_10_IU/mL. Retrospective testing at admission: HEV RNA = 5.4 log_10_IU/mL	Nosocomial transmission from case 1 (shared 2‐bedded space in the ICU; no physical contact)	Intubation and ventilation at ICU. Remdesivir, dexamethasone, tocilizumab, antibiotics	Ribavirin 400 mg twice daily for 24 weeks	Covid‐19: Transferred in ICU 7 days post‐admission. Progressively improved and transferred to medical ward on day 88. HEV: HEV RNA positivity below the lower limit of quantification (< 1.7 log_10_IU/mL) at week 12 of therapy. Lost to follow‐up
3 [12],	China, 2021	M, 69	High blood pressure, Chronic lymphocytic leukemia	On day 2 post‐admission: HEV IgG and IgM positive. On day 6 post‐admission: HEV RNA positive. SARS‐CoV‐2 detection 6 days post‐admission	Not documented	Nirmatrelvir/ritonavir and methylprednisolone	Polyene phosphatidylcholine capsules, S‐adenosylmethionine, then methylprednisolone on day 4 post‐admission, switched to dexamethasone until day 42	Day 60: SARS‐CoV‐2 RNA negative. Day 68: discharge from hospital in excellent clinical stage. One‐month post‐discharge: Negative HEV RNA
4, Present case	France, 2021	M, 50s	Marginal zone lymphoma	At admission, concurrently with admission for oxygen‐dependent pneumonia. Day 5 post‐admission: positive HEV RNA in blood (8.3 log_10_ IU/mL) and feces (7.5 log_10_IU/mL), Negative HEV IgG and IgM	Unknown	Oxygen therapy, dexamethasone, anakinra, and casirivimab‐imdevimab (combined with ceftriaxone then piperacillin/tazobactam)	Ribavirin (800 mg per day) for 3 months followed by a three‐month interruption due to ribavirin shortage. Then ribavirin reintroduction at a dosage of 800 mg per day for three months, ribavirin cessation for four months, then ribavirin administration at a dosage of 800 mg per day for six months, at a dosage of 1200 mg per day for six months, and at a dosage of 400 mg per day for 10 months. Thereafter the patient was lost to follow‐up	Negative SARS‐CoV‐2 RNA on day 5. Weaning from oxygen therapy on day 10 and hospital discharge on day 15. Negative HEV RNA in the blood on month 10 then positive again four months later. After ribavirin reintroduction for 22 months, HEV RNA remained weakly detectable in plasma, on five samples then the patient was lost to follow‐up

Abbreviations: HEV, hepatitis E virus; ICU, intensive care unit; IU, international unit.

## Author Contributions

Philippe Colson designed the study. All authors provided materials, data or analysis tools. All authors analyzed the data. Yacine Boucetta, Julien Andreani and Philippe Colson wrote the first draft of the manuscript. All authors reviewed and approved the final manuscript.

## Ethics Statement

The present study has been approved and registered on the Health Data Access Portal of Marseille public and university hospitals (Assistance Publique‐Hôpitaux de Marseille (AP‐HM)) under No. PADS24‐203 and has been approved by the Ethics and Scientific Committee of AP‐HM under No. CSE24‐203.

## Conflicts of Interest

PC is a scientific advisor for the BioSellal and Triber companies. The other authors have no conflicts of interest to declare. Funding sources had no role in the design and conduct of the study; collection, management, analysis, and interpretation of the data; and preparation, review, or approval of the manuscript.

## Data Availability

The data that support the findings of this study are openly available in GenBank at https://www.ncbi.nlm.nih.gov/genbank/, reference number ON276954.1, PQ878621.1.

## References

[1] N. Zhu , D. Zhang , W. Wang , et al., “A Novel Coronavirus From Patients With Pneumonia in China, 2019,” New England Journal of Medicine 382 (2020): 727–733, 10.1056/NEJMoa2001017. 31978945 PMC7092803

[2] N. Kamar , J. Izopet , N. Pavio , et al., “Hepatitis E Virus Infection,” Nature Reviews Disease Primers 3 (2017): 17086. 10.1038/nrdp.2017.86 29154369

[3] M. Kaba , P. Brouqui , H. Richet , et al., “Hepatitis E Virus Infection in Sheltered Homeless Persons, France,” Emerging Infectious Diseases 16 (2010): 1761–1763, 10.3201/eid1611.091890. 21029538 PMC3294504

[4] D. Cucinotta and M. Vanelli , “WHO Declares COVID‐19 a Pandemic,” Acta bio‐medica: Atenei Parmensis 91 (2020): 157–160, 10.23750/abm.v91i1.9397. 32191675 PMC7569573

[5] Z. Xu , S. Li , S. Tian , H. Li , and L. Kong , “Full Spectrum of COVID‐19 Severity Still Being Depicted,” Lancet 395 (2020): 947–948, 10.1016/S0140-6736(20)30308-1. PMC7133601 32066525

[6] E. Le Glass , V. T. Hoang , C. Boschi , et al., “Incidence and Outcome of Coinfections With SARS‐CoV‐2 and Rhinovirus,” Viruses 13 (2021): 2528, 10.3390/v13122528. 34960797 PMC8709236

[7] I. Aksamentov , C. Roemer , E. Hodcroft , and R. Neher , “Nextclade: Clade Assignment, Mutation Calling and Quality Control for Viral Genomes,” Journal of Open Source Software 6 (2021): 3773, 10.21105/joss.03773.

[8] P. Colson , P. E. Fournier , H. Chaudet , et al., “Analysis of SARS‐ CoV‐2 Variants From 24,181 Patients Exemplifies the Role of Globalization and Zoonosis in Pandemics,” Frontiers in Microbiology 12 (2022): 786233, 10.3389/fmicb.2021.786233. 35197938 PMC8859183

[9] P. Colson , P. Borentain , I. Ravaux , and S. Aherfi , “Hepatitis B Virus Genomics Knocking at the Door of Routine Diagnostic Laboratories,” Journal of Infectious Diseases 221, no. 6 (2020): 1026–1029, 10.1093/infdis/jiz544. 31628473

[10] D. B. Smith , J. Izopet , F. Nicot , et al., “Update: Proposed Reference Sequences for Subtypes of Hepatitis E Virus (Species Orthohepevirus A),” Journal of General Virology 101 (2020): 692–698, 10.1099/jgv.0.001435. 32469300 PMC7660235

[11] T. Lampejo , C. Curtis , S. Ijaz , et al., “Nosocomial Transmission of Hepatitis E Virus and Development of Chronic Infection: The Wider Impact of COVID‐19,” Journal of Clinical Virology 148 (2022): 105083, 10.1016/j.jcv.2022.105083. 35086023 PMC8785262

[12] C. Liu , D. Tang , J. Shi , G. Chen , and L. Gong , “Hepatitis E Virus and SARS‐CoV‐2 Co‐Infection in an Immunocompromised Patient: A Case Report,” Diagnostic Microbiology and Infectious Disease 110 (2024): 116471, 10.1016/j.diagmicrobio.2024.116471. 39079189

[13] C. Zhang , L. Shi , and F. S. Wang , “Liver Injury in COVID‐19: Management and Challenges,” Lancet Gastroenterology & Hepatology 5 (2020): 428–430, 10.1016/S2468-1253(20)30057-1. 32145190 PMC7129165

[14] N. Wanner , G. Andrieux , P. Badia‐I‐Mompel , et al., “Molecular Consequences of SARS‐CoV‐2 Liver Tropism,” Nature Metabolism 4, no. 3 (2022): 310–319, 10.1038/s42255-022-00552-6. PMC8964418 35347318

[15] P. Liptak , L. Nosakova , R. Rosolanka , L. Skladany , and P. Banovcin , “Acute‐On‐Chronic Liver Failure in Patients With Severe Acute Respiratory Syndrome Coronavirus 2 Infection,” World Journal of Hepatology 15 (2023): 41–51, 10.4254/wjh.v15.i1.41. 36744167 PMC9896507

[16] G. L. H. Wong , T. C. F. Yip , V. W. S. Wong , et al., “SARS‐CoV‐2 Viral Persistence Based on Cycle Threshold Value and Liver Injury in Patients With COVID‐19,” Open Forum Infectious Diseases 8 (2021): ofab205, 10.1093/ofid/ofab205. 34099979 PMC8135402

[17] S. K. Sarin , A. Choudhury , G. K. Lau , et al., “Pre‐Existing Liver Disease is Associated With Poor Outcome in Patients With SARS CoV2 Infection; The APCOLIS Study (APASL COVID‐19 Liver Injury Spectrum Study),” Hepatology International 14, no. 5 (2020): 690–700, 10.1007/s12072-020-10072-8. 32623632 PMC7334898

[18] M. Iavarone , R. D'Ambrosio , A. Soria , et al., “High Rates of 30‐day Mortality in Patients With Cirrhosis and COVID‐19,” Journal of Hepatology 73 (2020): 1063–1071, 10.1016/j.jhep.2020.06.001. 32526252 PMC7280108

[19] S. Chen , Y. Zhang , A. Ashuo , et al., “Combination of Spatial Transcriptomics Analysis and Retrospective Study Reveals Liver Infection of SARS‐COV‐2 is Associated With Clinical Outcomes of COVID‐19,” EBioMedicine 111 (2025): 105517, 10.1016/j.ebiom.2024.105517. 39709771 PMC11732063

[20] D. Ji , D. Zhang , T. Yang , et al., “Effect of COVID‐19 on Patients With Compensated Chronic Liver Diseases,” Hepatology International 14 (2020): 701–710, 10.1007/s12072-020-10058-6. 32734407 PMC7391917

[21] T. N. Chau , K. C. Lee , H. Yao , et al., “SARS‐Associated Viral Hepatitis Caused by a Novel Coronavirus: Report of Three Cases,” Hepatology 39 (2004): 302–310, 10.1002/hep.20111. 14767982 PMC7165792

[22] S. K. Satapathy , N. C. Roth , C. Kvasnovsky , et al., “Risk Factors and Outcomes for Acute‐on‐Chronic Liver Failure in COVID‐19: A Large Multi‐Center Observational Cohort Study,” Hepatology International 15 (2021): 766–779, 10.1007/s12072-021-10181-y. 33826042 PMC8024443

